# Reviewer acknowledgment

**DOI:** 10.1002/1348-9585.12371

**Published:** 2022-11-22

The Japan Society for Occupational Health expresses its sincere appreciation to the following volunteers for reviewing manuscripts for *Journal of Occupational Health* from October 1, 2021, to September 30, 2022.

Ae, Ryusuke (Japan)

Allison, Penelope J. (United States)

Amadi Obasi, Cecilia Nwadiuto (Nigeria)

Anami, Kunihiko (Japan)

Arakawa, Ritsuko (Japan)

Araki, Yoko (Japan)

Berthelsen, Hanne (Sweden)

Bocalini, Danilo (Brazil)

Chang, Wushou (Taiwan)

Cheng, Yawen (Taiwan)

Craddock, Tina (United States)

De Beer, Leon (South Africa)

Du, Tanghuizi (Japan)

Duarte, Joana (Sweden)

Ebara, Takeshi (Japan)

Eguchi, Hisashi (Japan)

Eguchi, Yasumasa (Japan)

Endo, Motoki (Japan)

Endo, Shota (Japan)

Ferri, Paola (Italy)

Fujii, Tomoko (Japan)

Fujino, Yoshihisa (Japan)

Fujisawa, Koichi (Japan)

Fujita, Takako (Japan)

Fujiwara, Takeshi (Japan)

Fukushima, Noritoshi (Japan)

Gatimu, Samwel (Kenya)

Griep, Rosane (Brazil)

Haga, Chiyori (Japan)

Hama, Sarkawt (United Kingdom of Great Britain and Northern Ireland)

Hamamura, Toshitaka (Japan)

Hara, Megumi (Japan)

Harris, Mark N. (Australia)

Hayashi, Toshio (Japan)

Heinonen, Noora (Finland)

Hidaka, Tomoo (Japan)

Hikichi, Hiroyuki (United States)

Hiraku, Yusuke (Japan)

Hirokawa, Kumi (Japan)

Hisanaga, Naomi (Japan)

Hori, Hajime (Japan)

Hori, Hikaru (Japan)

Horie, Seichi (Japan)

Ichiba, Masayoshi (Japan)

Ichihara, Gaku (Japan)

Ichihara, Sahoko (Japan)

Ikeda, Atsuko (Japan)

Ikegami, Kazunori (Japan)

Inaba, Ryoichi (Japan)

Irigoyen‐Otiñano, María (Spain)

Irokawa, Toshiya (Japan)

Ishimaru, Tomohiro (Japan)

Ishitake, Tatsuya (Japan)

Ito, Yuki (Japan)

Iwakiri, Kazuyuki (Japan)

Iwata, Hiroko (Japan)

Iwata, Toyoto (Japan)

Izumi, Hiroyuki (Japan)

Jang, Tae‐Won (Korea, the Republic of)

Jiang, Ying (Japan)

Kabe, Isamu (Japan)

Kajiki, Shigeyuki (Japan)

Kakamu, Takeyasu (Japan)

Kaltofen, Marco Paul Johann (United States)

Kamijima, Michihiro (Japan)

Kanamori, Satoru (Japan)

Kang, Mo‐Yeol (Korea, the Republic of)

Katayama, Akihiko (Japan)

Kawakami, Tsuyoshi (Switzerland)

Kawamura, Yoko (Japan)

Kawanami, Shoko (Japan)

Kawashima, Masatoshi (Japan)

Khalili, Arash (Iran, the Islamic Republic of)

Kido, Takamasa (Japan)

Kim, Inah (Korea, the Republic of)

Kingma, Idsart (Netherlands)

Kitamura, Hiroko (Japan)

Kiyono, Ken (Japan)

Kobayashi, Sumitaka (Japan)

Kobayashi, Tomoko (Japan)

Kobayashi, Yuka (Japan)

Kondo, Naoki (Japan)

Kozaki, Tomoaki (Japan)

Kubo, Tomohide (Japan)

Kudo, Yasushi (Japan)

Kumagai, Shinji (Japan)

Kuroda, Reiko (Japan)

Kurosawa, Hajime (Japan)

Kusama, Taro (Japan)

Kuwahara, Keisuke (Japan)

Lars, Van Tuin (Netherlands)

Lee, Wanhyung (Korea, the Republic of)

Li, Yongxin (China)

Lin, Ro‐Ting (Taiwan)

Lu, Ming‐Lun (United States)

Machida, Shuichi (Japan)

Mafune, Kosuke (Japan)

Massimi, Azzurra (Italy)

Masuda, Masashi (Japan)

Matsugaki, Ryutaro (Japan)

Michishita, Ryoma (Japan)

Miyagawa, Naoko (Japan)

Miyake, Yoshihiro (Japan)

Miyamoto, Toshiaki (Japan)

Miyauchi, Hiroyuki (Japan)

Momma, Haruki (Japan)

Mori, Koji (Japan)

Morimoto, Yasuo (Japan)

Morioka, Ikuharu (Japan)

Morita, Manabu (Japan)

Morita, Yusaku (Japan)

Mulkhan, Unang (Indonesia)

Murakami, Takahisa (Japan)

Muraki, Satoshi (Japan)

Murayama, Hiroshi (Japan)

Mustafaoglu, Rustem (Turkey)

Muto, Go (Japan)

Nagano, Chikage (Japan)

Nagata, Tomohisa (Japan)

Nakamura, Mieko (Japan)

Nishimura, Yoshito (Japan)

Nogawa, Kazuhiro (Japan)

Oda, Eiko (Japan)

Odagiri, Yuko (Japan)

Oe, Misari (Japan)

Ogami, Akira (Japan)

Ogawa, Masanori (Japan)

Ohta, Masanori (Japan)

Ojima, Jun (Japan)

Ojima, Toshiyuki (Japan)

Okazaki, Ryuji (Japan)

Omiya, Tomoko (Japan)

Oshio, Takashi (Japan)

Ota, Atsuhiko (Japan)

Otsuka, Yasumasa (Japan)

Owari, Yutaka (Japan)

Rabal‐Pelay, Juan (Spain)

Revilla, Josefa Angelie (Philippines)

Ruseski, Jane (United States)

Sado, Mitsuhiro (Japan)

Saengboonmee, Charupong (Thailand)

Saijo, Yasuaki (Japan)

Saito, Isao (Japan)

Saitoh, Hiroyuki (Japan)

Sakakibara, Keiko (Japan)

Sasaki, Natsu (Japan)

Sato, Yukihiro (Japan)

Sawada, Shinichi (Japan)

Shibata, Eiji (Japan)

Shibata, Nobuyuki (Japan)

Shibuya, Tomoaki (Japan)

Shimanoe, Chisato (Japan)

Shimura, Akiyoshi (Japan)

So, Rina (Japan)

Sugama, Atsushi (Japan)

Sugawara, Norio (Japan)

Sugiyama, Daisuke (Japan)

Sun, Rui (Netherlands)

Suwazono, Yasushi (Japan)

Tabuchi, Takahiro (Japan)

Tachi, Norihide (Japan)

Tahara, Hiroyuki (Japan)

Takahara, Ryuji (Japan)

Takahashi, Masaya (Japan)

Takahashi, Toru (Japan)

Takaya, Mitsutoshi (Japan)

Tamakoshi, Koji (Japan)

Tanaka, Rie (Japan)

Tani, Naomichi (Japan)

Tanihara, Shinichi (Japan)

Tateishi, Seiichiro (Japan)

Tatemichi, Masayuki (Japan)

Tatsumi, Yukako (Japan)

Theodoro, Heloísa (Brazil)

Togo, Fumiharu (Japan)

Tokizawa, Ken (Japan)

Tominaga, Maki (Japan)

Tomonaga, Taisuke (Japan)

Trépanier, Sarah‐Geneviève (Canada)

Tsuchiya, Masao (Japan)

Tsuno, Kanami (Japan)

Uchida, Mitsuo (Japan)

Ueno, Satoru (Japan)

Ueyama, Jun (Japan)

Uitti, Jukka (Finland)

Ukawa, Shigekazu (Japan)

Van Wijk, Charles (South Africa)

Vicente Herrero, Maria Teofila (Spain)

Videira‐Silva, Antonio (Portugal)

Vinnikov, Denis (Russian Federation)

Wada, Hiroo (Japan)

Wongrathanandha, Chathaya (Thailand)

Wang, Faming (China)

Wang, Rui‐Sheng (Japan)

Watai, Izumi (Japan)

Watanabe, Kazuhiro (Japan)

Watanabe, Sintaroo (Japan)

Weller, R. B. (United Kingdom of Great Britain and Northern Ireland)

Yamada, Keiko (Japan)

Yamamoto, Naofumi (Japan)

Yamauchi, Takashi (Japan)

Yasaci, Zeynal (Turkey)

Yoda, Takeshi (Japan)

Yokoyama, Sumi (Japan)

Yoshikawa, Etsuko (Japan)

Yoshioka, Eiji (Japan)

Zaitsu, Masayoshi (Japan)

